# Mitigation of Patulin in Fresh and Processed Foods and Beverages

**DOI:** 10.3390/toxins9050157

**Published:** 2017-05-11

**Authors:** J. David Ioi, Ting Zhou, Rong Tsao, Massimo F. Marcone

**Affiliations:** 1 Guelph Research and Development Centre, Agriculture and Agri-Food Canada, Guelph, ON N1G5C9, Canada; jioi@uoguelph.ca (J.D.I.); ting.zhou@agr.gc.ca (T.Z.); 2 Department of Food Science, University of Guelph, Guelph, Ontario, N1G 2W1, Canada; mmarcone@uoguelph.ca

**Keywords:** patulin, mycotoxin, mitigation, decontamination, food and beverage, processing

## Abstract

Patulin is a mycotoxin of food safety concern. It is produced by numerous species of fungi growing on fruits and vegetables. Exposure to the toxin is connected to issues neurological, immunological, and gastrointestinal in nature. Regulatory agencies worldwide have established maximum allowable levels of 50 µg/kg in foods. Despite regulations, surveys continue to find patulin in commercial food and beverage products, in some cases, to exceed the maximum limits. Patulin content in food can be mitigated throughout the food processing chain. Proper handling, storage, and transportation of food can limit fungal growth and patulin production. Common processing techniques including pasteurisation, filtration, and fermentation all have an effect on patulin content in food but individually are not sufficient safety measures. Novel methods to remove or detoxify patulin have been reviewed. Non-thermal processing techniques such as high hydrostatic pressure, UV radiation, enzymatic degradation, binding to microorganisms, and chemical degradation all have potential but have not been optimised. Until further refinement of these methods, the hurdle approach to processing should be used where food safety is concerned. Future development should focus on determining the nature and safety of chemicals produced from the breakdown of patulin in treatment techniques.

## 1. Introduction

**Figure 1:**
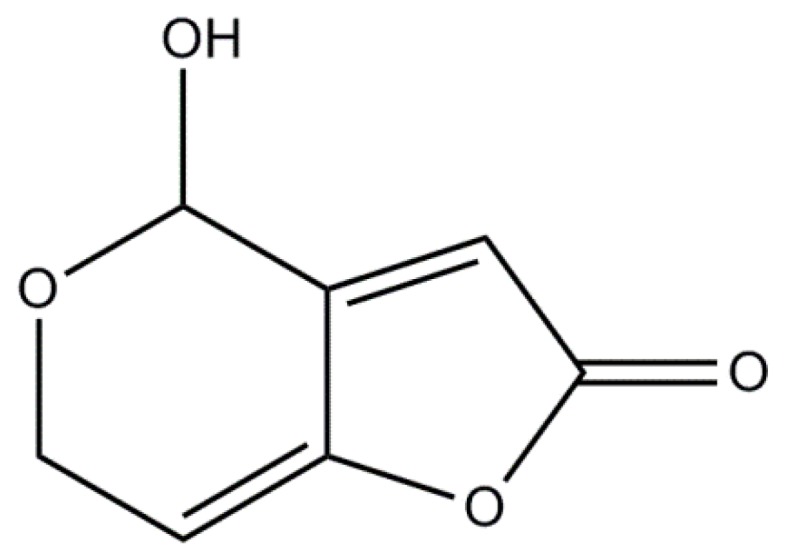
Chemical structure of patulin.

Mycotoxins are toxic secondary metabolites produced by fungi that present a potential hazard regrading food safety. Patulin is a mycotoxin and is known to be produced by more than 60 species of fungi belonging to greater than 30 genera [1,2]. Although typically associated with *Penicillium expansum*, patulin is also known to be produced by other fungi, including *P. claviforme*, *P. urticae*, *P. patulum*, *Aspergillus clavatus*, *A. giganteus*, *Byssoclamys fulva*, *B. nivea*, and *Alternaria alternata* [3]. Chemically speaking, patulin (4-hydroxy-4-H-furo[3,2-c]pyran-2[6H]-one) is an unsaturated heterocyclic lactone with a molecular weight of 154 (Figure 1) [4,5]. Discovered in the 1940s, patulin was initially investigated for its potential as an antibiotic due to its strong activity against gram-positive and gram-negative bacteria such as *Mycobacterium tuberculosis* [6] and more than 75 other different bacterial species [7]. In the 1960s, it was found to be toxic not only to bacterial cells but to animal and plant cells as well and was subsequently reclassified as a mycotoxin [8].

Patulin has a broad spectrum of toxicities that include both acute and chronic effects. Some examples include congestion and edema of blood vessels and tissues [9]. Formation of sarcomas has been observed when large doses of the mycotoxin were injected into animals, causing concerns of carcinogenicity in humans [10]. Other effects seen in animals include teratogenicity, liver, spleen and kidney damage, lung and brain edema, and immune system toxicity [11]. In humans, the primary reported acute symptoms include gastrointestinal issues, nausea, and vomiting, but there is no conclusive evidence as to the nature of the chronic effects [3]. The LD_50_ of patulin ranges from 15 to 25 mg/kg and is dependent on the characteristics of exposure as well as the route of ingestion [5]. Patulin is a highly reactive molecule, able to interact with proteins to form intramolecular and intermolecular crosslinks with specific amino acids, causing it to behave as an enzyme inhibitor [12,13]. It has also been shown that patulin can form intermolecular links with DNA molecules [14]. These properties may explain the reported teratogenic and carcinogenic effects. No reports are available on the possible toxicity of patulin due to inhalation of the toxin in powdered form [15]. Children are more at risk for toxicities from patulin as they often consume more potentially contaminated products. Information taken from a study by the USDA has shown that children have a very high consumption of apple products as compared to adults [16]. During the first year of life, children were found to consume on average 6.4 g/kg body weight/day of apples while adults consumed only 0.4 g/kg body weight/day [16]. This means that it is of particular importance to be cautious of the potential danger that patulin and other mycotoxins present in baby foods. 

Due to the potential negative health effects of consuming patulin, regulatory agencies from around the world have instituted limits regarding the maximum amount of patulin that can be in food products. Many organizations such as Health Canada, The United States Food and Drug Administration, and the Codex Committee on Food Additives and Contaminants have all set limits of 50 µg/kg patulin [17,18,19]. The World Health Organization has suggested a limit of 0.4 µg/kg body weight and the European Union has set a much lower maximum limit of 25 µg/kg for solid products and 10 µg/kg for any food marketed towards infants [20,21]. 

Despite the presence of these regulations, patulin continues to be found in food products around the world. Table 1 summarizes the patulin contamination that has been quantified in various food products. Patulin is typically associated with apples and apples products; however it has also been found in other fruits such as pears, figs, and tomatoes [22,23,24]. Scientific surveys have also discovered patulin contamination in vegetables such as bell peppers, grains like wheat, rice and corn, and some cheeses [25,26,27].

In some cases patulin has been found in commercial food products exceeding regulatory limits. An examination of apple and pear products in Tunisia for patulin contamination found that 50% of samples were contaminated [28]. The level of patulin ranged from 2 μg/kg to 889 μg/kg, with 22% of contaminated samples exceeding the limit for the toxin set by the European Union. Another study based in Turkey found that dried figs contained patulin in levels as high as 151 μg/kg [23].

Other studies have surveyed food products for the presence of spores of patulin producing fungi. The concern is that, should fungi capable of producing mycotoxins survive processing, they may under the right circumstances continue to produce patulin later. Fungi such as *P. expansum*, *B. nivea*, and *A. terreus* have been isolated from cereals and a variety of fruits [29]. Strains of patulin producing fungi have been found in other products such as peanuts, pecans, and hazelnuts [30]. 

The presence of fungi does not necessarily indicate the presence of patulin within a food. Likewise the absence of visible fungal growth is not a guarantee of safety. The mycotoxin has been shown to diffuse away from mold in food products. In apples, patulin has been found 1–2 cm away from the infected flesh [31,32]. Similar diffusion was seen in pears in a later study [33]. In tomatoes, likely due to the lower viscosity of their interiors, patulin can diffuse throughout the entirety of the fruit [31]. The same study also found that patulin can diffuse through wheat based products by as much as 3–4 cm. 

Given the potential toxicity of patulin and the continued occurrence of it in commercial food products, there has been research done to look at the effect of different treatments on patulin. This review will examine the methods used to mitigate the threat of patulin in food through the commercial food chain with a focus on techniques devised for the specific function of mycotoxin reduction. 

## 2. Pre-Processing Control of Patulin

The degree of patulin contamination in a food product can be managed at all levels in the food processing chain. There are common pre-processing steps that are completed regardless of the end product. These can have a significant effect on the patulin content of the finished food. These steps include storage, removal of the fungi from fresh fruit and vegetables, and the application of fungicides.

### 2.1. Storage

Prevention of fungal growth is the first step in the mitigation of mycotoxins. The conditions that foods face directly after harvest and before processing can have a large effect on the final quality of the produce. *P. expansum* is known to possess psychrotrophic characteristics; it is able to grow and produce patulin under refrigeration temperatures [47]. This fungus is able to produce patulin between 4 and 25 °C for 20 to 90 days [48]. The implication is that refrigerated storage is only suitable for relatively short periods of storage. The Food and Agriculture Organization of the United Nations (FAO) suggests keeping storage to <10 °C or to store fresh produce for <48 h in order to prevent the risk of patulin. Another study determined that the 48 h mark is only important for produce stored at 20 °C or higher (open deck storage); it found that in refrigerated storage the 48 h mark was not a critical time slot [49]. 

The use of a modified atmosphere is a second control option used for the storage of food products. The application of a high carbon dioxide and/or nitrogen atmosphere with low oxygen content has been shown to be a potential means of controlling mold growth and rot in apples [50,51]. Apples packaged in polyethylene have shown high degrees of inhibition of fungal growth and patulin production, while polypropylene is not an effective inhibiting material [52]. Without the use of a modified atmosphere, polyethylene limited growth by 68% and toxin production by 99.5%. Inhibition was further increased with the use of a modified atmosphere with CO_2_ in a dose dependent manner on both patulin production and fungal growth. 

### 2.2. Fungicides

Another means for controlling mold growth and mycotoxins in the field and in storage is the application of fungicides. There are a large number of fungicides that have been shown to possess varying levels of effectiveness. Benzimidazole fungicides used to be a common form of post-harvest treatment for fruit to deter fungal growth but have seen a steep decline in use due to increased fungal resistance [53]. Ripening makes fruits more sensitive to contamination by mold; ripened fruit put into storage had a higher risk of contamination in the absence of fungicide use. In contrast, when treated with fungicide, the more ripened fruit had smaller amounts of fungal contamination than the unripened ones, suggesting that the efficacy of fungicides is dependent on the characteristics of the fruit and the degree of ripening at the onset of storage [54]. Fludioxonil is another conventional fungicide that has been found to be effective in controlling *Penicillium* growth in apples [55]. While the use of conventional fungicides is an effective control measure, increased regulatory and health concerns and fungal resistance to the fungicides have led to research into finding alternative control agents [47]. A naturally occurring volatile compound trans-2-hexenal has been shown to be an effective fumigant in controlling *P. expansum* growth and patulin reduction in apples during storage [56]. A 3% solution of sodium hypochlorite (NaOCl) also effectively inhibited the growth of a number of fungi, including *P. expansum*, *A. alaternata*, and *Fusarium* sp., on apples [57]. Essential oils such as lemon and orange oils were also tested in the same study, and it was found that the production of patulin by *P. expansum* in apples was completely inhibited by a 0.2% solution of lemon oil and by >90% with the use of 0.05% lemon oil and 0.2% orange oil solutions. 

### 2.3. Physical Removal of Fungi and Infected Tissue

The overall quality of food products is highly dependent on the quality of the raw ingredients. Ingredients with a high concentration of mycotoxin contamination will inherently cause a high level of contamination in the finished product. It is a recommended practice to sort out any damaged produce prior to processing and storage [58]. This not only decreases the patulin content in the finished product but also helps to reduce the possibility of cross contamination. Unfortunately the highest quality produce are typically sorted out for sale as fresh produce, whereas it is common to use bruised damaged fruit or even windfalls for the creation of juices or purees [59,60]. While this is purely a choice to lower the cost and make use of lower quality fruits, it can decrease the safety of food products. There are other steps that can be taken in order to reduce the final patulin content and increase product quality other than sorting out infected fruit. These will typically either take the form of a washing or trimming process. 

Washing typically involves either immersion into a tumbling water tank/bath or application of a high pressure water stream [61]. The primary purpose of the washing step is to remove debris, including dirt, plant matter, bugs, and mold/fungi [60]. Patulin is a water soluble molecule, and, by including a washing step, a portion of the patulin content in produce can be solubilized and removed [62]. Studies on the effects of washing treatments on the patulin level in apples found that this was one of the most critical stages of processing and could remove up to 54% of patulin from infected apples [61,63]. Researchers found that use of a high pressure water spray was more effective than a rotary wash tub as the spray would also aid in the physical removal of infected tissue for an improved reduction of both patulin and pathogenic fungi. The efficacy of washing can vary, ranging from 10% to 100% reductions [64]. 

Research has also been done into examining whether a chemical wash solution would be more effective than water at reducing patulin. Both the United States Food and Drug Administration (USFDA) and the Canadian Food Inspection Agency (CFIA) have recommended the use of a 100–150 ppm chlorine wash solution for processed fruit and vegetable products [65,66]. The use of a chlorine solution is primarily for the purpose of reducing microbial levels and not for the destruction of patulin. The effectiveness of chlorine as an antimicrobial is highly dependent on pH [67]. Research has been conducted to compare the use of a chlorine solution as opposed to pure water to reduce patulin content in apples [64], but, other than a larger impact on mold/fungi counts, no significant difference was found in patulin reduction by chlorine treatment. Chlorine levels need to be closely monitored as chlorine may cause the possible formation of chemical by-products and increased wear and corrosion of the equipment [68]. Other options for treating fruit include chlorine dioxide [68], hydrogen peroxide [69], acetic acid vapour [70], ozonated water [58,71], calcium salts [72], and electrolyzed oxidizing water [73]. A 200 ppm NaOCl solution inhibited the growth of *P. expansum* but was not able to completely prevent the production of patulin [67]. The same study found that the use of an acetic acid solution of 2–5% for >1 min was able to completely inhibit the growth of mold and prevent the production of patulin. 

While an effective technique for removing patulin, a washing step alone is not sufficient to guarantee the safety of fresh food products. There is also the potential for the wash water to spread the patulin and cause cross contamination. Moreover, if not properly handled and disposed of, the wash solution containing patulin becomes a potential source of contamination for the entire processing facility. Washing is not a sufficient standalone mitigation technique and should be applied as a carefully monitored critical control point with other patulin reduction methods following it. 

Another means of patulin reduction by fungi removal is the trimming of tissue from produce that is infected with fungi or is otherwise damaged. Regarding apples, patulin has been shown to concentrate around the area of infection and thus the removal of just this section of the apple will significantly reduce its patulin content [47]. Trimming damaged tissue was found to be capable of removing more than 93–99% of the total patulin content from apples [74]. 

Trimming of the fruit is an economically useful means of patulin reduction as, unlike sorting, only small portions of the material is discarded. The danger of this method is that the contaminated material must be handled properly and disposed of following its removal from the apple. This allows for possible cross contamination as well as a potential increased cost to processors. The other limitation is that, as previously discussed, patulin can diffuse from the infected area to other areas of the food products [31]. For products such as tomatoes, trimming will have no significant effect on patulin reduction. 

## 3. Effect of Processing Steps on Patulin

Commercially sold food products have been found to contain patulin, in some cases exceeding the regulatory limit. As has been discussed, pre-processing methods can have a significant impact on the reduction of the patulin content. There is a limit to the efficacy of such techniques, and in instances of high patulin contents these methods are not able to reduce patulin to the regulatory limit. Studies have been conducted to determine the effect of conventional processing on patulin, with the intention of combining these techniques with pre-processing methods in an integrated system to increase safety. 

### 3.1. Clarification/Filtration

Clarification or filtration techniques are commonly used in the manufacture of fruit juices. The purpose of these steps is primarily to remove solid particles such as pectins or proteins from solution [61]. This category of processing techniques has been studied for their potential to also reduce the patulin content from liquid apple products.

A system of depectinisation, clarification (with gelatine and bentonite), and filtration using a rotary vacuum pre-coat filter could reduce patulin content by 39% [61]. The depectinisation step caused negligible change to the patulin content, and the clarification step was found to be responsible for the majority of the reduction in patulin. A different study examining filtration media found that bentonite gave a patulin reduction of 8.5% and diatomaceous earth only 3.4% [75]. This same study also examined the use of centrifugation either alone or combined with other filtration methods. This type of separation works based on the difference in force applied to substances as determined by their individual masses [76]. Centrifugation alone reduced patulin content by 20.5% [75].

The removal of patulin by activated carbon/charcoal as a filtration medium has been extensively studied [77,78,79,80]. An early work compared stirring powdered activated carbon in the juice with using an activated carbon filter screen [77] and found that both methods were equally effective in reducing patulin in apple juice by 98.15–100%. It should to be noted that follow up studies did not find such high levels of reduction by activated carbon [78]. Nevertheless, activated carbon was found to be the most effective clarification means to reduce patulin, although it was also found to cause the highest reduction in pH, colour, sugar content, and possibly other nutrients. Different types of activated carbon showed different effects on patulin removal. Steam activated carbon was more effective than chemically activated carbon [44]. The same study also suggested that more activated carbon was required to reduce patulin in juices with higher solids contents.

Enzymatic depectinisation is a common juice/concentrate clarification method that uses pectinase enzymes to break down the pectin that surrounds protein particles in the solution, causing the protein to sediment out [60]. Depectinisation has been studied for its potential to also remove patulin from apple juice with varying results. Enzyme treatment only reduced patulin content by <5% in previous studies [61,75,80]; however, a study using apple juice samples from an industrial processing facility found that the use of enzymatic depectinisation removed 28% of the patulin content [81]. It is theorized that the patulin removal ability of this method is due to patulin binding to the solid particles that are removed from the solution [75]. Given these varying results, enzyme treatment does not appear to be a practical means of controlling patulin contamination.

Microfiltration and ultrafiltration have been used in recent years as an additional means of liquid product clarification. Membrane filtration separates molecules based on the molecular weight of solutes suspended in a solution [82]. Microfiltration typically filters out molecules in the size range of 0.1–2 µm, and ultrafiltration in the range of 0.001–0.1 µm. These types of systems are advantageous over conventional clarifying methods in that they cut down on enzyme consumption and the use of other filtering aids and are readily adapted to a continuous flow process [81]. The two methods are distinguished primarily by difference in membrane pore size; thus the maximum molecular weights they will let through [76]. Both have been studied for their ability to filter out patulin from apple juice [61,78]. Microfiltration could reduce the patulin content by 20.1% at different stages of apple juice processing [81]. Ultrafiltration was less effective in patulin removal from apple juice, with a 3–12% reduction [61,78]. The ability of membrane filtration to remove patulin is likely dependent on its binding to larger molecules that can be removed by the membranes rather than filtering out patulin itself [81]. This could explain the disparity of results, as the individual experimental parameters and particular composition of the apple juices used may have had a greater effect on the results than the difference in filter size. 

### 3.2. Heat Treatment

Heat treatments such as pasteurization and distillation are common preservation methods in food processing [76]. Pasteurization is a mild heat treatment used to extend the shelf life and increase the safety of foods by destroying detrimental microorganisms. Evaporation and distillation are processes that use heat to remove water and/or volatile components from liquid foods. 

The use of thermal treatments to reduce the risk of patulin has been questioned due to its resistance to heat [74,83,84,85]. Pasteurization has historically shown differing and contrasting results with regard to patulin reduction. These are summarized briefly in Table 2. Pasteurization for 20 min at 80 °C of apple juice spiked with patulin was found to cause a reduction of up to 50% [86]; however a later study reported that patulin was stable at 80 °C for 30 min in apple juice, and a more severe treatment of 120 °C for 30 min was required in order to achieve patulin reduction [87]. A high temperature short time (HTST) system was found to cause greater reduction in patulin (18.8% reduction at 90 °C) compared to a batch pasteurization treatment [88]. Re-examination of the two methods found that longer time pasteurization of 20 min at 90°C and 100 °C could lead to 18.81% and 25.99% patulin reduction, respectively [89]. Conflicting results continue to be generated by different investigators when comparing HTST and batch pasteurization [81,89]. 

Distillation, on the other hand, was found to reduce patulin by 24% in apple juice; however this was likely due to the degradation of patulin into breakdown products by heat as opposed to removal as a volatile phase [87]. This was confirmed in a later study [90]. 

The ability of heat treatments to remove patulin is highly dependent on the treatment parameters as well as the characteristics of the apple product. It has been indicated that, for a juice that has an initial patulin level equal to or greater than 200 μg/kg, no variation of heat treatment can reduce the patulin to below the regulated level of 50 μg/kg [89]. Other components of the food matrices may also play a role in how patulin is affected. As in filtration, it is possible that the presence of other large molecules such as fibre, protein, or sugars may cause binding to patulin and an increased resistance to destruction [81]. The pH of the product may also have had an effect on patulin reduction. Patulin is stable in acidic conditions; it has shown resistance to heat at a pH range of 3.5–5.5 at 125 °C [85]. 

### 3.3. Fermentation

Fermentation is a process in which yeast converts sugars into alcohols, gases, and/or acids [76]. Numerous studies have been reported on the contamination of unfermented apple juice by patulin; however few surveys have documented patulin contamination in alcoholic cider [93,94]. Yeast fermentation was found to be able to reduce patulin by up to 90% [95]. Another study used eight strains of yeast and three different types of fermentation processes to assess the effect on patulin in apple juice spiked with 15 mg/L patulin and found that all treatments were able to reduce the patulin content by 99% over a two week fermentation period [96]. Similarly, in an examination of the ability of three different strains of *Saccharomyces cerevisiae* to degrade patulin in apple juice, it was found that all three were effective at reducing the patulin content during fermentation but were ineffective during periods of aerobic growth [94]. This suggests that the reduction of patulin seen is a result of degradation by fermentation as opposed to adsorbing to the yeast cells themselves. This study also examined the breakdown products of patulin by fermentation and whether they were adsorbed by the yeast cells [94]. They found that numerous decomposition products were generated by fermentation and that they remained in the juice after fermentation. Of note, one of these products was *E*-ascadiol, which is a mycotoxin itself, though considered to be less toxic than patulin [97]. 

## 4. Patulin Reduction Techniques

Conventional processing has been shown to have an effect on patulin content in food products [47,61]. The extent to which the patulin content can be reduced by these means is unclear, with results dependent on the parameters and the initial patulin content [22,37]. This suggests that further treatment may be required to reduce the mycotoxin content. Methods of processing specifically for the reduction of patulin have been proposed to replace traditional methods or for inclusion in the production process as part of a hurdle approach. These include biological, chemical, and physical methods to either bind patulin or degrade it. 

### 4.1. Biological Control Agents

Biological control of patulin refers to any method which uses microorganisms to reduce the patulin content in a product or to prevent the production of patulin. These methods differ from fermentation in that they do not necessarily contribute to the characteristics or chemical properties of the food; they are studied for the purpose of patulin reduction. This control method falls under two general categories based on the mode of patulin reduction; detoxification and adsorption. Detoxification refers to processes that chemically modify the mycotoxin to inactivate or reduce toxicity, while adsorption refers to the means by which the mycotoxin is bound and removed from solution. Similar to the fermentative removal of patulin by the yeast *S. cerevisiae*, microorganisms such as lactic acid bacteria (LAB), other yeasts, and fungi have been investigated for their ability to degrade patulin in aqueous solution [98,99,100,101,102]. Microorganisms, including LAB and yeast cells have also been found to be capable of patulin reduction in food products through the mechanism of adsorption onto their cell walls [103,104,105,106,107,108]. 

LAB are microorganisms of significance due to their high usage in the food industry as both a means to process food and as additives. They are used as probiotics in maintaining gastrointestinal health and have been shown to offer protection against some toxic compounds [109]. Research has found that significant amounts of patulin were removed by 10 strains of LAB, resulting in a 47 to 80% reduction of patulin [106]. The result also suggests that this reduction of patulin by LAB is highly strain specific. The increased surface area and cell wall volume of the bacteria showed a higher ability to adsorb patulin from aqueous solution [108]. Functional groups including C-O, OH, and NH were found to be involved in adsorbing patulin, suggesting that polysaccharides and proteins that are rich in these groups may play important roles. The effect of cell viability on degree of patulin removal has also been assed [107]. The highest reduction of patulin was achieved by a strain of *Bifidobacterium bifidum* at 51.1% for viable cells and 54.1% for nonviable cells. 

Yeast cells or cell wall components have also been studied for their ability to bind patulin. Two types of inactivated yeast powder were examined for their capacity to adsorb the mycotoxin [105]. Patulin was reduced to below 4.6 µg/kg after 36 h. Cell powders of eight or 10 yeast strains were shown to be able to reduce patulin by greater than 50% over a 24 h period [104]. The ability of two *Enterococcus faecium* strains to remove patulin from aqueous solution has also been investigated [103]. The strains were able to remove between 15% and 45% of the patulin over a 48 h period. The viability of the strains did not have a significant effect on the ability to remove patulin. It was also found that patulin forms a stable complex with the bacteria, and the patulin-bacteria complexes could reasonably be maintained through further processing.

Microorganisms have also been studied for their ability to detoxify patulin. Two strains of *Metschnikowia pulcherrima* yeast were able to degrade patulin in a liquid media spiked with 5, 7.5, 10, and 15 μg/mL patulin [101]. It was found that one of the yeast strains was able to reduce patulin levels by 100% within 48 h and the other within 72 h at all concentrations. No patulin was found in the cell walls after degradation, suggesting that the ability of these yeast strains to remove patulin is unrelated to cell wall binding, as was the case in the other form of biological control of patulin. Researchers also noted that the presence of patulin did not influence the concentration of yeast cells during growth, suggesting that the yeast was immune to the toxic effects of the mycotoxin. It has not been determined whether all yeast strains possess patulin resistance. However, using a pre-treatment of low amounts of patulin prior to fermentation, it was found that patulin resistance and degradation abilities could be induced in *Sporobolomyces* sp. cells [102]. A variety of yeast strains have been examined for their ability to degrade patulin. The yeast *P. ohmeri* was able to degrade more than 83% of patulin after two days at 25 °C, and after 15 days patulin was degraded below the detectable limit [99]. *S. cerevisiae* degraded 96% of patulin in apple juice that had an initial concentration of 4.5 µg/mL after 6 d at 25 °C. However only 90% was degraded when the initial patulin content was 7.0 µg/mL, suggesting that the rate of degradation is concentration dependent. A strain of marine yeast, *Kodameae ohmeri*, has been reported to have a high tolerance to patulin and to have the ability to significantly reduce patulin content in apple juice. It has been suggested that the ability of yeast to detoxify patulin is enzymatic in nature [110]. It was found that the yeast *Rhodosporidium paludignum* could significantly reduce the patulin content in apples and pears. A potential hazard concerning the use of *R. paludignum* has also been noted; however the application of this yeast in high concentrations actually increased the patulin concentration in infected fruits [111]. This is possibly caused by a triggering of stress responses of patulin-producing fungi. Another concern with patulin degradation is the potential toxicity of the breakdown products. While not all of the degradation products have been assessed, some have been identified as *E*-ascladiol, *Z*-ascladiol, and deoxypatulinic acid [98,100,112]. *E*-ascladiol and *Z*-ascladiol have been found to exhibit no signs of toxicity towards human cell lines derived from the intestinal tract, kidney, liver, and immune system [113].

### 4.2. Chemical Additives

Several methods of chemical degradation of patulin have been proposed. Some of these are novel methods, while others are additives used for other purposes in apple production but that have found subsequent use as patulin reducers. Of the variety of chemicals studied, the most promising include ascorbic acid, ammonia, potassium permanganate, sulfur dioxide, ozone, and some of the B vitamins [95,114,115,116,117].

Ascorbic acid and ascorbate (Vitamin C) have been studied to reduce patulin in apple products. Slight reductions of patulin by ascorbic acid have been reported, with one study noting only 5% losses after 3 h and 36% after 44 h [115]. Similarly, the degradation of patulin during storage was observed; the patulin content of apple juice with ascorbic acid added was decreased by 70% but only by 30% in juice without added ascorbic acid after a 34 d period [118].

The degradation of patulin by ammoniation and by oxidation with potassium permanganate in an acidic and a basic environment has also been studied as a control measure [115]. Both treatments were effective and able to reduce patulin by more than 99.9% in a standard aqueous solution. Under acidic conditions, treatment with potassium permanganate was found to produce potentially mutagenic and harmful compounds, limiting its potential for use in foodstuffs. 

Patulin has been shown to be unstable in the presence of sulfur containing compounds [119]. For this reason there has been study on the effect of sulfur dioxide to degrade patulin in solution. One study found that a solution with 200 ppm of sulfur dioxide was able to reduce patulin by 12% after 24 h and 90% after two days [95]. A later study found that just 100 ppm sulfur dioxide could reduce patulin content by 50% in 15 min. The differences noted here could be attributed to differences in the composition of the sample solutions, as interfering components may have been present. Patulin is also thought to react with a number of other sulfur compounds to produce less toxic compounds [9,120]. It has been shown that patulin will form adducts with a number of sulfur containing compounds such as cysteine, *N*-acetylcysteine, and glutathione [12]. This is because patulin has a strong affinity for binding covalently to sulfhydryl groups as well as amino, thiol, and NH_2_ groups [121]. While still possessing some toxicity, it has been determined that these adducts are 100 times less toxic than patulin itself [122]. 

Ozone is a strong oxidant, capable of reacting with numerous chemical groups and is thought to be able to detoxify patulin [116,121,123]. Patulin treated with a 10% solution of ozone degraded from 5 ppm to below detectable levels in 15 s [116]. Metal ions in general did not affect patulin degradation by ozone oxidation although iron and manganese both significantly reduced the effect. Ozone alone was able to degrade patulin by up to 98% in 1 min [123]. Ozone treatment is highly effective and it does not have a significant effect on the quality parameters of food products [121]. 

Among thiamine hydrochloride, pyridoxine hydrochloride, and calcium d-pantothenate, the latter i.e. calcium d-pantothenate was the most effective at reducing patulin, being able to reduce it by up to 94.3% over six months with no significant loss in quality characteristics, compared to 35.8% with no addition of any substance [117]. While effective, this length of time may be impractical for many products and the toxicity of the adducts formed requires further study. 

### 4.3. Physical Treatments

Patulin is known to be resistant to degradation by heat treatment [85]. Furthermore some of the treatments used in apple processing that have been suggested as possibilities for the reduction of patulin are known to have a negative effect on some of the quality characteristics of the food product such as pH, clarity, colour, sugars, and °Brix [78,79,91]. Development in the field of non-thermal food processing techniques has opened up the potential for unconventional processing methods to play a role in apple processing and the reduction of patulin.

#### 4.3.1. Ultraviolet Radiation

Ultraviolet (UV) radiation is an approved non-thermal method for the preservation of fruit juices in both Canada and the United States [124,125]. Typically used for the destruction of microorganisms, it has also been studied as a means of degrading patulin [126,127]. UV radiation with an exposure range of 14.2–99.4 mJ/cm^2^ on apple cider was able to cause reductions ranging from 9.4 to 43.4% with higher exposures causing higher patulin reductions [128]. No loss of chemical components or sensory properties was found including pH, °Brix, and total acids. A later study on the effect of UV radiation (253.7 nm) on patulin in apple juice, apple cider, and a model aqueous solution showed that it was highly effective in all but apple cider [127,129]. It was suggested that apple cider might contain components that are interfering with the breakdown reaction. The increased turbidity of apple cider is considered to hamper the action of UV. The use of filtration/clarification processes therefore can increase the effectiveness of this technique on apple cider. Other studies have shown that the effect of UV radiation can be significantly hindered by the presence of large amounts of ascorbic acid [126]. Ascorbic acid is a common additive to apple juice for its anti-browning and antioxidant properties [130]. In order for UV radiation to be an effective treatment, any addition of ascorbic acid would have to occur afterwards. The particular wavelength of UV light used was also shown to affect patulin reduction [131]. It was determined that patulin reduction in apple juice or cider was most effective at 222 nm as opposed to 254 and 282 nm. No significant changes to pH, soluble solids, or the colour of the apple juice were found during this treatment. Despite this apparent lack of change to other components of apple juice, a trained sensory panel found that UV radiation treated juice was significantly different from conventionally treated juice [126]. Further research should examine not only the effect of UV on patulin but also on flavour compounds. 

#### 4.3.2. Pulsed Light

The use of pulsed light is another processing technique that has been proposed for the destruction of patulin in food products [132]. Pulsed light is a non-thermal food preservation technique that involves the use of short (1 µs–0.1 s) bursts of broad spectrum light with wavelengths ranging from 200 to 1100 nm [133]. Patulin was degraded by pulsed light in MclIvaine buffer, apple juice, and apple puree [132]. Treatment with pulsed light was able to reduce the patulin content by 85–95% in the buffer, 22% in the juice, and 51% in the apple puree. The effectiveness was not dependent on the initial patulin content. 

#### 4.3.3. High Hydrostatic Pressure

High hydrostatic pressure processing (HPP) is a non-thermal food processing method originally designed for the reduction of microorganisms that has also been studied as a means of reducing mycotoxins in foods [134]. HPP is a food preservation treatment that uses high pressure to inactivate microorganisms and proteins [135,136]. HPP treatment has been found to reduce up to 56.24% of patulin in apple juice contaminated with 100 ppb of the mycotoxin, depending on the operating conditions [134]. Pressures ranged from 30 to 500 Mpa, and temperatures ranged from (30–50 °C). No clear trend as to the optimal pressure/temperature combination has been concluded, suggesting that further study is required to refine this technology. A higher pressure at 600 MPa for 300 s was found to reduce patulin in juice by 31% [137]. It has been shown that HPP primarily works on hydrophobic and electrostatic interactions, not the covalent ones found in patulin molecules [138]. The reductions in patulin content have been attributed to the formation of adducts with compounds containing sulphhydryl groups such as glutathione or cysteine [134,139]. These adducts have been shown to be 100 times less toxic than patulin itself [122].

## 5. Conclusions

Patulin is a mycotoxin of threat to human health. While the precise nature of the long term effects in humans are uncertain, the evidence from animal cases is sufficient to justify concern. A number of regulatory agencies have laid out limitations as to the allowable patulin content in food products. Despite these regulations, patulin maintains a presence in foods produced around the world. Means of removing or detoxifying patulin in food are necessary considerations in the processing chain to increase safety. 

A combination of temperature control, the use of modified atmosphere storage, and the application of fungicides can significantly decrease fungal growth and the production of patulin in fresh fruits. However not every storage facility has access to all of these technologies, and there are issues with the over use of chemical fungicides. Improving the quality of the fruit to be processed by means of washing, trimming, and sorting are all very useful means for controlling patulin. These processes may not be economically feasible for all producers as they also significantly increase raw material waste. The waste itself presents a contamination issue and requires special consideration for handling and disposal. Typical pre-processing techniques are not sufficient and further patulin reduction is necessary. Conventional processing methods using heat, fermentation, and/or chemical binders have similarly been shown to have an effect on patulin. However the evidence is inconclusive as to the actual efficacy of these techniques and so cannot be relied upon to guarantee safety. Furthermore, there is concern with the potential toxicity of compounds produced by the degradation of patulin by some of these methods. Most of these products have yet to be identified, and some that have been identified are known to be toxic. 

Due to the resistance of patulin to acidic conditions and heat treatment, alternative methods have been proposed for the removal or detoxification of patulin using modified or novel processing techniques. For these methods to be considered for this purpose they must be safe and effective. The FAO has requirements for a decontamination process that state that the method must do the following:

1. Destroy, inactivate, or otherwise remove the mycotoxins.
2. Not leave or create any products that possess toxic/mutagenic/carcinogenic properties.
3. Be practical in so far as it is technologically and economically feasible.
4. Prevent the re-occurrence of mycotoxins by destroying any fungal spores or mycelium.

The development of non-thermal physical processing techniques has led to some promising possibilities for techniques to reduce the patulin content of food products. UV and pulsed light radiation can effectively remove patulin from solution; however they may also destroy beneficial components of the food. HPP can be an effective means of patulin degradation; however the optimal conditions have yet to be determined and may depend in part on the properties of the food product. Regarding the FAO requirements, the primary concern for any of these treatments is that current research is inadequate to fully explain the mechanism of action and the potential toxicity of the breakdown products. These methods would also require highly specialized equipment, which represents a major cost factor and may not be allowed for use in food products. 

Chemical degradation of patulin is another potential solution, given that the method is easily accommodated into traditional processing streams. However, the use of chemical agents solely for the purpose of mycotoxin reduction is currently not permitted, though many of the chemicals listed here can also serve other purposes; for example, ascorbic acid (Vitamin C) is commonly used to prevent oxidation or browning [60,140]. Despite this potential, further research is still required to validate some of the inconsistencies found and to determine the optimal processing conditions. The exact nature of the reactions and, more importantly, the reaction products for many of these cases are still unknown, which provides some health and safety concerns. 

Biologically based methods can eliminate or reduce the patulin content in food products. Both enzymatic degradation and the adsorptive removal of patulin by microorganisms like LAB or yeast have a significant impact on patulin content. Furthermore, it has been found that the application of biological control methods causes no significant impact to the juice quality characteristics of °Brix, acidity, colour, and clarity [104]. Various microorganisms have the ability to either detoxify or bind to patulin in an aqueous environment, though it is unclear what the optimal processing conditions are. It has been shown that strain, pH, temperature, incubation time, concentration of microorganisms, and patulin level all play an important role, but further study is required [106]. Some breakdown products have been identified, but not all are known [98,100,112]. More work is required to more fully understand the processes involved and to determine how to safely incorporate these methods into food processing. 

There are numerous methods that have an effect on fungi and patulin; however there does not appear to be any singular method that can reliably prevent patulin in food products. Instead the most practical course of action appears to be the application of the hurdle approach to food safety. That is to say that multiple safety checks and measures should be implemented throughout the entire growth, harvest, and production cycle. Work on the refinement of these techniques to determine the optimal processing conditions is necessary to increase the practicality of usage. The main concern at this point and the area to which future research focus must be turned is in understanding and controlling the degradation compounds that are produced by a number of these methods. 

## Figures and Tables

**Table 1:** Recent surveys of the occurrence of patulin in food commodities.

Food Commodity	Location	Range (µg/kg)	Positive (%)	Reference
Apples	Spain	0	0	[34]
Apples	Portugal	1–70.6	ND	[24]
Apples	United States of America	8.8–417.6	40.8	[35]
Figs	Turkey	39.3–151.6	ND	[23]
Tomatoes	Portugal	21.29	ND	[24]
Tomatoes	Belgium	ND	10.8	[25]
Bell Peppers	Belgium	ND	11.4	[25]
Hazelnuts	Turkey	16.6–92.4	ND	[36]
Cereal Based Foods	Portugal	0–4.5	75	[26]
Apple Juice	Italy	1.6–55.4	47	[37]
Apple Juice	Turkey	7–376	100	[38]
Apple Juice	Brazil	3–7	3	[39]
Apple Juice	Tunisia	2–889	64	[28]
Apple Juice	Portugal	1.2–42	41	[40]
Apple Juice	Belgium	2.5–39	81	[41]
Apple Juice	Spain	0–36.5	45	[42]
Apple Juice	South Korea	9.9–30.9	12.5	[43]
Apple Juice	Spain	2.5–6	7.1	[30]
Apple Juice	South Africa	5–45	24	[44]
Apple Juice	United States of America	8.8–2700.4	22.7	[35]
Apple Puree	Argentina	22–221	50	[22]
Apple Puree	Portugal	1.2–5.7	7	[40]
Apple Puree	Spain	0–50.3	13	[42]
Apple Puree	Italy	1.92	-	[45]
Apple Puree	South Africa	5–20	35	[44]
Apple Products	Argentina	17–39	16	[22]
Apple Products	China	1.2–94.7	12.6	[46]
Pear Products	Argentina	25	17	[22]
Pear Products	Italy	0.79	ND	[45]
Tomato Products	Italy	7.15	ND	[45]
Fruit Jam	Tunisia	2–554	20	[28]
Pear Juice	Tunisia	5–231	47.6	[28]
Orange Juice	South Korea	9.9–30.9	8	[43]
Grape Juice	South Korea	5.2–14.5	17	[43]
Semi-hard cheese	Italy	15–460	28	[27]

ND = No data available.

**Table 2:** Patulin degradation during heat treatment of liquid food products.

Processing Temperature (°C)	Processing Time (min)	Initial PAT (µg/kg)	PAT Reduction (%)	Reference
80	20	4	55	[86]
80	30	ND	NS	[87]
90	0.17	96.5	13.4	[80]
90	0.17	20	19	[88]
90	0.5	433	39.6	[81]
90	7	1500	60	[32]
90	10	140	12.1	[91]
90	20	220	18.8	[89]
90	20	1000	NS	[92]
100	20	220	26	[89]

NS = No significant reduction in patulin found; ND = No data available; PAT = patulin.

## References

[1] Barug D., Bhatnagar D., Van Egmond H.P., van der Kamp J.W., Van Osenbruggen W.A., Visconti A. (2006). The Mycotoxin Factbook.

[2] Lai C., Fuh Y., Shih D. (2000). Detection of mycotoxin patulin in apple juice. J. Food Drug Anal..

[3] Drusch S., Ragab W. (2003). Mycotoxins in fruits, fruit juices, and dried fruits. J. Food Prot..

[4] Stott W., Bullerman L.B. (1975). Patulin: A mycotoxin of potential concern in foods. J. Food Prot..

[5] Yang J., Li J., Jiang Y., Duan X., Qu H., Yang B., Chen F., Sivakumar D. (2014). Natural occurrence, analysis, and prevention of mycotoxins in fruits and their processed products. Crit. Rev. Food Sci. Nutr..

[6] Paterson R.R., Venancio A., Lima N. (2004). Solutions to *Penicillium* taxonomy crucial to mycotoxin research and health. Res. Microbiol..

[7] Ciegler A., Detroy R., Lillehoj E. (1971). Patulin, penicillic acid, and other carcinogenic lactones. Microb. Toxins.

[8] Puel O., Galtier P., Oswald I.P. (2010). Biosynthesis and toxicological effects of patulin. Toxins.

[9] Ciegler A., Vesonder R., Jackson L. (1977). Production and biological activity of patulin and citrinin from *Penicillium expansum*. Appl. Environ. Microbiol..

[10] Dickens F., Jones H. (1961). Carcinogenic activity of a series of reactive lactones and related substances. Br. J. Cancer.

[11] Llewellyn G.C., McCay J.A., Brown R.D. (1998). Immunological evaluation of the mycotoxin patulin in female B6C3F_1_ mice. Food Chem. Toxicol..

[12] Fliege R., Metzler M. (2000). Electrophilic properties of patulin. Adduct structures and reaction pathways with 4-bromothiophenol and other model nucleophiles. Chem. Res. Toxicol..

[13] Baert K., Devlieghere F., Flyps H., Oosterlinck M., Ahmed M.M., Rajkovic A., Verlinden B., Nicolai B., Debevere J., De Meulenaer B. (2007). Influence of storage conditions of apples on growth and patulin production by *Penicillium expansum*. Int. J. Food Microbiol..

[14] Schumacher D.M., Müller C., Metzler M., Lehmann L. (2006). DNA-DNA cross-links contribute to the mutagenic potential of the mycotoxin patulin. Toxicol. Lett..

[15] Fung F., Clark R.F. (2004). Health effects of mycotoxins: A toxicological overview. J. Toxicol. Clin. Toxicol..

[16] Plunkett L., Turnbull D., Rodricks J., Guzelian P., Henry C., Olin S. (1992). Differences between adults and children affecting exposure assessment. Similarities and Differences between Children and Adults, Implications for Risk Assessment.

[17] CODEX, Codex Alimentarius Commission (2003). Maximum Level for Patulin in Apple Juice and Apple Juice Ingredients and Other Beverages.

[18] Food and Drug Administration (FDA) (2004). Compliance policy guidance for fda staff. Sec. 510.150 Apple juice, apple juice concentrates, and apple juice products—Adulteration with patulin. Compliance Policy Guide.

[19] Health Canada, Health Canada (2014). Canadian standards for various chemical contaminants in foods. Food and Drug Regulations.

[20] World Health Organization (1995). Evaluation of certain food additives and contaminants. Tech. Rep. Ser..

[21] European Commission (2006). Commission regulation (EC) No 1881/2006 of 19 december 2006 setting maximum levels for certain contaminants in foodstuffs. Off. J. Eur. Union.

[22] Funes G.J., Resnik S.L. (2009). Determination of patulin in solid and semisolid apple and pear products marketed in argentina. Food Control.

[23] Karaca H., Nas S. (2006). Aflatoxins, patulin and ergosterol contents of dried figs in turkey. Food Addit. Contam..

[24] Cunha S.C., Faria M.A., Pereira V.L., Oliveira T.M., Lima A.C., Pinto E. (2014). Patulin assessment and fungi identification in organic and conventional fruits and derived products. Food Control.

[25] Van de Perre E., Jacxsens L., Van Der Hauwaert W., Haesaert I., De Meulenaer B. (2014). Screening for the presence of patulin in molded fresh produce and evaluation of its stability in the production of tomato products. J. Agric. Food Chem..

[26] Assuncao R., Martins C., Dupont D., Alvito P. (2016). Patulin and ochratoxin a co-occurrence and their bioaccessibility in processed cereal-based foods: A contribution for portuguese children risk assessment. Food Chem. Toxicol..

[27] Pattono D., Grosso A., Stocco P.P., Pazzi M., Zeppa G. (2013). Survey of the presence of patulin and ochratoxin a in traditional semi-hard cheeses. Food Control.

[28] Zouaoui N., Sbaii N., Bacha H., Abid-Essefi S. (2015). Occurrence of patulin in various fruit juice marketed in tunisia. Food Control.

[29] Deshpande S.S. (2002). Handbook of Food Toxicology.

[30] Marín S., Mateo E.M., Sanchis V., Valle-Algarra F.M., Ramos A.J., Jiménez M. (2011). Patulin contamination in fruit derivatives, including baby food, from the spanish market. Food Chem..

[31] Rychlik M., Schieberle P. (2001). Model studies on the diffusion behavior of the mycotoxin patulin in apples, tomatoes, and wheat bread. Eur. Food Res. Technol..

[32] Taniwaki M., Hoenderboom C., De Almeida Vitali A., Firoa M. (1992). Migration of patulin in apples. J. Food Prot..

[33] Laidou I.A., Thanassoulopoulos C.C., Liakopoulou-Kyriakides M. (2001). Diffusion of patulin in the flesh of pears inoculated with four post-harvest pathogens. J. Phytopathol..

[34] Marin S., Morales H., Hasan H.A., Ramos A.J., Sanchis V. (2006). Patulin distribution in Fuji and golden apples contaminated with *Penicillium expansum*. Food Addit. Contam..

[35] Harris K., Bobe G., Bourquin L. (2009). Patulin surveillance in apple cider and juice marketed in michigan. J. Food Prot..

[36] Ekinci R., Otag M., Kadakal C. (2014). Patulin & ergosterol: New quality parameters together with aflatoxins in hazelnuts. Food Chem..

[37] Spadaro D., Ciavorella A., Frati S., Garibaldi A., Gullino M.L. (2007). Incidence and level of patulin contamination in pure and mixed apple juices marketed in italy. Food Control.

[38] Gokmen V., Acar J. (1998). Incidence of patulin in apple juice concentrates produced in turkey. J. Chromatogr. A.

[39] Iha M.H., Sabino M. (2008). Incidence of patulin in brazilian apple-based drinks. Food Control.

[40] Barreira M.J., Alvito P.C., Almeida C.M.M. (2010). Occurrence of patulin in apple-based-foods in portugal. Food Chem..

[41] Tangni E.K., Theys R., Mignolet E., Maudoux M., Michelet J.Y., Larondelle Y. (2003). Patulin in domestic and imported apple-based drinks in belgium: Occurrence and exposure assessment. Food Addit. Contam..

[42] Pique E., Vargas-Murga L., Gomez-Catalan J., Lapuente J., Llobet J.M. (2013). Occurrence of patulin in organic and conventional apple-based food marketed in catalonia and exposure assessment. Food Chem. Toxicol..

[43] Cho M.S., Kim K., Seo E., Kassim N., Mtenga A.B., Shim W.-B., Lee S.-H., Chung D.-H. (2010). Occurrence of patulin in various fruit juices from South Korea: An exposure assessment. Food Sci. Biotechnol..

[44] Leggott N., Shephard G. (2001). Patulin in south african commercial apple products. Food Control.

[45] Sarubbi F., Formisano G., Auriemma G., Arrichiello A., Palomba R. (2016). Patulin in homogenized fruit’s and tomato products. Food Control.

[46] Yuan Y., Zhuang H., Zhang T., Liu J. (2010). Patulin content in apple products marketed in northeast China. Food Control.

[47] De Souza Sant’Ana A., Rosenthal A., de Massaguer P.R. (2008). The fate of patulin in apple juice processing: A review. Food Res. Int..

[48] Taniwaki M., Bleinroth E., De Martin Z. (1989). Bolores produtores de patulina em macã e suco industrializado. Colet. Inst. Tecnol. Alimentos.

[49] Morales H., Marin S., Centelles X., Ramos A.J., Sanchis V. (2007). Cold and ambient deck storage prior to processing as a critical control point for patulin accumulation. Int. J. Food Microbiol..

[50] Johnsonn D., Stow J., Dover C. (1993). Prospect for the control of fungal rotting in cox’s orange pippin apples by low oxygen and low ethylene storage. Acta Hortic..

[51] Paster N., Huppert D., Barkai-Golan R. (1995). Production of patulin by different strains of *Penicillium expansum* in pear and apple cultivars stored at different temperatures and modified atmospheres. Food Addit. Contam..

[52] Moodley R.S., Govinden R., Odhav B. (2002). The effect of modified atmospheres and packaging on patulin production in apples. J. Food Prot..

[53] Rosenberger D. Control of *Penicillium Expansum* During Apple Harvest Storage. Proceedings of the Patulin Technical Symposium.

[54] Morales H., Marin S., Rovira A., Ramos A.J., Sanchis V. (2007). Patulin accumulation in apples by *Penicillium expansum* during postharvest stages. Lett. Appl. Microbiol..

[55] Errampalli D. (2004). Effect of fludioxonil on germination and growth of *Penicillium expansum* and decay in apple cvs. Empire and gala. Crop Prot..

[56] Neri F., Mari M., Menniti A.M., Brigati S., Bertolini P. (2006). Control of *Penicillium expansum* in pears and apples by trans-2-hexenal vapours. Postharvest Biol. Technol..

[57] Hasan H.A. (2000). Patulin and aflatoxin in brown rot lesion of apple fruits and their regulation. World J. Microbiol. Biotechnol..

[58] CODEX, Codex Alimentarius Commission (2003). Code of Practice for the Prevention and Reduction of Patulin Contamination in Apple Juice and Apple Juice Ingredients in other Beverages.

[59] Moake M.M., Padilla-Zakour O.I., Worobo R.W. (2005). Comprehensive review of patulin control methods in foods. Compr. Rev. Food Sci. Food Saf..

[60] Root W.H., Barrett D.M., Barrett D.M., Somogyi L., Ramaswamy H. (2005). Apples and apple processing. Processing Fruits.

[61] Acar J., Gokmen V., Taydas E.E. (1998). The effects of processing technology on the patulin content of juice during commercial apple juice concentrate production. Z. Lebensm. Unters. Forsch. A.

[62] Cole R., Jarvis B.B., Schweikert M.A. (2003). Handbook of Secondary Fungal Metabolites.

[63] Sydenham E., Vismer H., Marasas W., Brown N., Schlechter M., van der Westhuizen L., Rheeder J. (1995). Reduction of patulin in apple juice samples—Influence of initial processing. Food Control.

[64] Jackson L.S., Beacham-Bowden T., Keller S.E., Adhikari C., Taylor K.T., Chirtel S.J., Merker R.I. (2003). Apple quality, storage, and washing treatments affect patulin levels in apple cider. J. Food Prot..

[65] Food and Drug Administration (FDA), Food Safety Initiative Staff, HFS-32, U.S. Food and Drug Administration (1998). Guide to Minimize Microbial Food Safety Hazards for Fresh Fruits and Vegetables.

[66] Canadian Food Inspection Agency (CFIA), Canadian Food Inspection Agency (2009). Code of Practice for Minimally Processed Ready-To-Eat Vegetables.

[67] Chen L., Ingham H., Ingham S.C. (2004). Survival of *Penicillium expansum* and patulin production on stored apples after wash treatments. J. Food Sci..

[68] Roberts R.G., Reymond S.T. (1994). Chlorine dioxide for reduction of postharvest pathogen inoculum during handling of tree fruits. Appl. Environ. Microbiol..

[69] Baldry M. (1983). The bactericidal, fungicidal and sporicidal properties of hydrogen peroxide and peracetic acid. J. Appl. Bacteriol..

[70] Sholberg P., Haag P., Hocking R., Bedford K. (2000). The use of vinegar vapor to reduce postharvest decay of harvested fruit. Hortic. Sci..

[71] Spotts R., Cervantes L. (1992). Effect of ozonated water on postharvest pathogens of pear in laboratory and packinghouse tests. Plant Dis..

[72] Conway W., Lanisiewicz W., Klein I., Sams C. (1999). Strategy for combining heat treatment, calcium infiltration, and biological control to reduce postharvest decay of “gala” apples. Hortic. Sci..

[73] Okull D., LaBorde L. (2004). Activity of electrolyzed oxidizing water against *Penicillium expansum* in suspension and on wounded apples. J. Food Sci..

[74] Lovett J., Thompson R., Boutin B. (1975). Patulin production in apples stored in a controlled atmosphere. J. Assoc. Off. Anal. Chem..

[75] Bisseur J., Permaul K., Odhav B. (2001). Reduction of patulin during apple juice clarification. J. Food Prot..

[76] Fellows P.J. (2009). Food Processing Technol.

[77] Sands D.C., McIntyre J.L., Walton G.S. (1976). Use of activated charcoal for the removal of patulin from cider. Appl. Environ. Microbiol..

[78] Gökmen V., Artık N., Acar J., Kahraman N., Poyrazoğlu E. (2001). Effects of various clarification treatments on patulin, phenolic compound and organic acid compositions of apple juice. Eur. Food Res. Technol..

[79] Kadakal C., Nas S. (2002). Effect of activated charcoal on patulin, fumaric acid, and some other properties of apple juice. Nahr. Food.

[80] Kadakal C., Sebahattin N., Poyrazoğlu E.S. (2002). Effect of commercial processing stages of apple juice on patulin, fumaric acid and hydroxymethylfurfural (HMF) levels. J. Food Qual..

[81] Welke J.E., Hoeltz M., Dottori H.A., Noll I.B. (2009). Effect of processing stages of apple juice concentrate on patulin levels. Food Control.

[82] Fukumoto L., Delaquis P., Girard B. (1998). Microfiltration and ultrafiltration ceramic membranes for apple juice clarification. J. Food Sci..

[83] Wiesner B. (1942). Bactericidal effects of *Aspergillus clavatus*. Nature.

[84] Heatley N., Philpot F. (1947). The routine examination for antibiotic produced by moulds. J. Gen. Microbiol..

[85] Lovett J., Peeler J. (1973). Effect of ph on the thermal destruction kinetics of patulin in aqueous solution. J. Food Sci..

[86] Scott P., Somers E. (1968). Stability of patulin and penicillic acid in fruit juices and flour. J. Agric. Food Chem..

[87] Kubacki S. The analysis and occurrence of patulin in apple juice. Proceedings of the 6th International IUPAC Symposium on Mycotoxins Phycotoxins.

[88] Wheeler J.L., Harrison M.A., Koehler P.E. (1987). Presence and stability of patulin in pasteurized apple cider. J. Food Sci..

[89] Kadakal C., Nas S. (2003). Effect of heat treatment and evaporation on patulin and some other properties of apple juice. J. Sci. Food Agric..

[90] Kryger R.A. (2001). Volatility of patulin in apple juice. J. Agric. Food Chem..

[91] Janotová L., Čížková H., Pivoňka J., Voldřich M. (2011). Effect of processing of apple puree on patulin content. Food Control.

[92] Woller R., Majerus P. (1982). Patulin in obsterzeugnissen-egenschaften, bildung und vorkommen. Flussiges Obst.

[93] Harwig J., Chen Y., Kennedy P., Scott P. (1973). Occurrence of patulin and patulin producing strains of *Penicillium expansum* in natural rots of apples in canada. J. Can. Inst. Food Sci. Technol..

[94] Moss M.O., Long M.T. (2002). Fate of patulin in the presence of the yeast *Saccharomyces cerevisiae*. Food Addit. Contam..

[95] Burroughs L. (1977). Stability of patulin to sulfur dioxide and to yeast fermentation. J. Assoc. Off. Anal. Chem..

[96] Stinson E.E., Osman S.F., Huhtanen C.N., Bills D.D. (1978). Disappearance of patulin during alcoholic fermentation of apple juice. Appl. Environ. Microbiol..

[97] Suzuki T., Takeda M., Tanabe H. (1971). A new mycotoxin produced by *Aspergillus clavatus*. Chem. Pharm. Bull..

[98] Ricelli A., Baruzzi F., Solfrizzo M., Morea M., Fanizzi F.P. (2007). Biotransformation of patulin by *Gluconobacter oxydans*. Appl. Environ. Microbiol..

[99] Coelho A., Celli M., Sataque Ono E., Hoffmann F., Pagnocca F., Garcia S., Sabino M., Harada K., Wosiacki G., Hirooka E. (2008). Patulin biodegradation using *Pichia ohmeri* and *Saccharomyces cerevisiae*. World Mycotoxin J..

[100] Fuchs S., Sontag G., Stidl R., Ehrlich V., Kundi M., Knasmuller S. (2008). Detoxification of patulin and ochratoxin a, two abundant mycotoxins, by lactic acid bacteria. Food Chem. Toxicol..

[101] Reddy K.R., Spadaro D., Gullino M.L., Garibaldi A. (2011). Potential of two *metschnikowia pulcherrima* (yeast) strains for in vitro biodegradation of patulin. J. Food Prot..

[102] Ianiri G., Pinedo C., Fratianni A., Panfili G., Castoria R. (2017). Patulin degradation by the biocontrol yeast *sporobolomyces* sp. Is an inducible process. Toxins.

[103] Topcu A., Bulat T., Wishah R., Boyaci I.H. (2010). Detoxification of aflatoxin B1 and patulin by *enterococcus faecium* strains. Int. J. Food Microbiol..

[104] Yue T., Dong Q., Guo C., Worobo R.W. (2011). Reducing patulin contamination in apple juice by using inactive yeast. J. Food Prot..

[105] Guo C., Yue T., Hatab S., Yuan Y. (2012). Ability of inactivated yeast powder to adsorb patulin from apple juice. J. Food Prot..

[106] Hatab S., Yue T., Mohamad O. (2012). Reduction of patulin in aqueous solution by lactic acid bacteria. J. Food Sci..

[107] Hatab S., Yue T., Mohamad O. (2012). Removal of patulin from apple juice using inactivated lactic acid bacteria. J. Appl. Microbiol..

[108] Wang L., Yue T., Yuan Y., Wang Z., Ye M., Cai R. (2015). A new insight into the adsorption mechanism of patulin by the heat-inactive lactic acid bacteria cells. Food Control.

[109] Knasmüller S., Steinkellner H., Hirschl A.M., Rabot S., Nobis E.C., Kassie F. (2001). Impact of bacteria in dairy products and of the intestinal microflora on the genotoxic and carcinogenic effects of heterocyclic aromatic amines. Mutat. Res..

[110] Zhu R., Yu T., Guo S., Hu H., Zheng X., Karlovsky P. (2015). Effect of the yeast *Rhodosporidium paludigenum* on postharvest decay and patulin accumulation in apples and pears. J. Food Prot..

[111] Zhu R., Feussner K., Wu T., Yan F., Karlovsky P., Zheng X. (2015). Detoxification of mycotoxin patulin by the yeast *Rhodosporidium paludigenum*. Food Chem..

[112] Castoria R., Mannina L., Duran-Patron R., Maffei F., Sobolev A.P., De Felice D.V., Pinedo-Rivilla C., Ritieni A., Ferracane R., Wright S.A. (2011). Conversion of the mycotoxin patulin to the less toxic desoxypatulinic acid by the biocontrol yeast *Rhodosporidium kratochvilovae* strain LS11. J. Agric. Food Chem..

[113] Tannous J., Snini S.P., El Khoury R., Canlet C., Pinton P., Lippi Y., Alassane-Kpembi I., Gauthier T., El Khoury A., Atoui A. (2016). Patulin transformation products and last intermediates in its biosynthetic pathway, *E*- and *Z*-ascladiol, are not toxic to human cells. Arch. Toxicol..

[114] Brackett R.E., Marth E.H. (1979). Ascorbic acid and ascorbate cause disappearance of patulin from buffer solutions and apple juice. J. Food Prot..

[115] Fremy J.M., Castegnaro M.J., Gleizes E., De Meo M., Laget M. (1995). Procedures for destruction of patulin in laboratory wastes. Food Addit. Contam..

[116] McKenzie K.S., Sarr A.B., Mayura K., Bailey R.H., Miller D.R., Rogers T.D., Norred W.P., Voss K.A., Plattner R.D., Kubena L.F. (1997). Oxidative degradation and detoxification of mycotoxins using a novel source of ozone. Food Chem. Toxicol..

[117] Yazici S., Velioglu Y.S. (2002). Effect of thiamine hydrochloride, pyridoxine hydrochloride and calcium-d-pantothenate on the patulin content of apple juice concentrate. Nahrung/Food.

[118] Drusch S., Kopka S., Kaeding J. (2007). Stability of patulin in a juice-like aqueous model system in the presence of ascorbic acid. Food Chem..

[119] Pohland A., Allen R. (1970). Stability studies with patulin. J. AOAC.

[120] Cavallito C., Bailey J. (1944). Preliminary note on the inactivation of antibiotics. Science.

[121] Wu T.S., Liao Y.C., Yu F.Y., Chang C.H., Liu B.H. (2008). Mechanism of patulin-induced apoptosis in human leukemia cells (HL-60). Toxicol. Lett..

[122] Lindroth S., von Wright A. (1990). Detoxification of patulin by adduct formation with cysteine. J. Environ. Pathol. Toxicol. Oncol..

[123] Karaca H., Sedat Velioglu Y. (2009). Effects of some metals and chelating agents on patulin degradation by ozone. Ozone Sci. Eng..

[124] Food and Drug Administration (FDA), U.S. Food and Drug Administration (2000). Irradiation in the Production, Processing and Handling of Food.

[125] Health Canada Ultraviolet Light Treatment of Apple Juice/Cider Using the Cidersure 3500. http://www.hc-sc.gc.ca/fn-an/gmf-agm/appro/dec85_rev_nl3-eng.php.

[126] Assatarakul K., Churey J.J., Manns D.C., Worobo R.W. (2012). Patulin reduction in apple juice from concentrate by UV radiation and comparison of kinetic degradation models between apple juice and apple cider. J. Food Prot..

[127] Tikekar R.V., Anantheswaran R.C., LaBorde L.F. (2014). Patulin degradation in a model apple juice system and in apple juice during ultraviolet processing. J. Food Process. Preserv..

[128] Dong Q., Manns D.C., Feng G., Yue T., Churey J.J., Worobo R.W. (2010). Reduction of patulin in apple cider by UV radiation. J. Food Prot..

[129] Zhu Y., Koutchma T., Warriner K., Shao S., Zhou T. (2012). Kinetics of patulin degradation in model solution, apple cider and apple juice by ultraviolet radiation. Food Sci. Technol. Int..

[130] Sapers G.M., Hicks K.B., Philips J.G., Garzarella L., Pondish D.L., Matulaitis R.M., McCormack T.J., Sondey S.M., Seib P.A., El-Atawy Y.S. (1989). Control of enzymatic browning in apple with ascorbic acid derivatives, polyphenol oxidase inhibitors, and complexing agents. J. Food Sci..

[131] Zhu Y., Koutchma T., Warriner K., Zhou T. (2014). Reduction of patulin in apple juice products by UV light of different wavelengths in the UVC range. J. Food Prot..

[132] Funes G.J., Gómez P.L., Resnik S.L., Alzamora S.M. (2013). Application of pulsed light to patulin reduction in mcilvaine buffer and apple products. Food Control.

[133] Gomez-Lopez V., Ragaert P., Debevere J., Devlieghere V. (2007). Pulsed light for food decontamination: A review. Trends Food Sci. Technol..

[134] Avsaroglu M.D., Bozoglu F., Alpas H., Largeteau A., Demazeau G. (2015). Use of pulsed-high hydrostatic pressure treatment to decrease patulin in apple juice. High Press. Res..

[135] San Martin M.F., Barbosa-Canovas G.V., Swanson B.G. (2002). Food processing by high hydrostatic pressure. Crit. Rev. Food Sci. Nutr..

[136] Rendueles E., Omer M.K., Alvseike O., Alonso-Calleja C., Capita R., Prieto M. (2011). Microbiological food safety assessment of high hydrostatic pressure processing: A review. LWT Food Sci. Technol..

[137] Hao H., Zhou T., Koutchma T., Wu F., Warriner K. (2016). High hydrostatic pressure assisted degradation of patulin in fruit and vegetable juice blends. Food Control.

[138] Patterson M.F. (2005). Microbiology of pressure-treated foods. J. Appl. Microbiol..

[139] Schebb N.H., Faber H., Maul R., Heus F., Kool J., Irth H., Karst U. (2009). Analysis of glutathione adducts of patulin by means of liquid chromatography (HPLC) with biochemical detection (BCD) and electrospray ionization tandem mass spectrometry (ESI-MS/MS). Anal. Bioanal. Chem..

[140] Park D.L., Troxell T.C. (2002). U.S. Perspective on mycotoxin regulatory issues. Adv. Exp. Med. Biol..

